# Community transmission of monkeypox in the United Kingdom, April to May 2022

**DOI:** 10.2807/1560-7917.ES.2022.27.22.2200422

**Published:** 2022-06-02

**Authors:** Roberto Vivancos, Charlotte Anderson, Paula Blomquist, Sooria Balasegaram, Anita Bell, Louise Bishop, Colin S Brown, Yimmy Chow, Obaghe Edeghere, Isaac Florence, Sarah Logan, Petra Manley, William Crowe, Andrew McAuley, Ananda Giri Shankar, Borja Mora-Peris, Karthik Paranthaman, Mateo Prochazka, Cian Ryan, David Simons, Richard Vipond, Chloe Byers, Nicholas A. Watkins, UKHSA Monkeypox Incident Management team, Will Welfare, Elizabeth Whittaker, Claire Dewsnap, Allegra Wilson, Yvonne Young, Meera Chand, Steven Riley, Susan Hopkins, Monkeypox Incident Management Team, Andre Charlett, Thomas Finnie, Helen McAuslane, Browne Weeple, Helen Fifer, Katy Sinka, David Edwards, Jamie Lopez-Bernal, Tommy Rampling, Andrew Lee, Dominic Mellon, Merav Kliner, Nick Young, Sophia Makki, Suzanne Coles, Wendi Shepherd, Victoria Latham, Ruby Tabor, Alice Graham, JinMin Yuan, Neil MacDonald, Amoolya Vusirikala, Thomas Ma, Kristine Cooper, Maria Saavedra-Campos

**Affiliations:** 1 UK Health Security Agency, London, England, United Kingdom; 2 NIHR Health Protection Research Unit in Emerging and Zoonotic Infections, UKHSA, England, United Kingdom; 3 NIHR Health Protection Research Unit in Gastrointestinal Infections, UKHSA, England, United Kingdom; 4 NIHR Health Protection Research Unit in Healthcare Acquired Infections and Antimicrobial Resistance, UKHSA, England, United Kingdom; 5 University College London Hospitals NHS Trust, London, England, United Kingdom; 6 Health and Social Care Northern Ireland, Antrim, Northern Ireland, United Kingdom; 7 Public Health Scotland, Edinburgh, Scotland, United Kingdom; 8 Public Health Wales, Cardiff, Wales, United Kingdom; 9 Imperial College Healthcare NHS Trust, London, England, United Kingdom; 10 Members of the UKHSA Monkeypox Incident Management team are listed under Collaborators; 11 The British Association for Sexual Health & HIV, England & Wales, United Kingdom

**Keywords:** Monkeypox, monkeypox virus, orthopoxvirus, transmission, viruses, zoonoses, United Kingdom

## Abstract

Between 7 and 25 May, 86 monkeypox cases were confirmed in the United Kingdom (UK). Only one case is known to have travelled to a monkeypox virus (MPXV) endemic country. Seventy-nine cases with information were male and 66 reported being gay, bisexual, or other men who have sex with men. This is the first reported sustained MPXV transmission in the UK, with human-to-human transmission through close contacts, including in sexual networks. Improving case ascertainment and onward-transmission preventive measures are ongoing.

Monkeypox is an emerging zoonotic infection caused by monkeypox virus (MPXV), which in the past has been primarily detected in West and Central Africa. The incubation period of monkeypox can be up to 21 days [1]. In the United Kingdom (UK) all previous seven cases ever reported were either imported, or household or healthcare contacts of imported cases [2-5]. In this report, we describe an ongoing outbreak of MPXV infections in the UK detected since the beginning of May 2022 affecting people without documented history of travel to endemic countries.

## Outbreak description

The outbreak cases are currently grouped into three distinct incidents. First, an isolated case was imported from Nigeria, an endemic country for MPXV, and laboratory confirmed by PCR on 7 May 2022 [6]. Of 116 contacts identified including healthcare workers, none developed MPXV infection at the end of their 21-day follow-up period.

Second, a separate household cluster was then detected on 12 May 2022, with two laboratory-confirmed cases and one case who had already clinically resolved (no laboratory confirmation). The onset of symptoms of the first case in the cluster was on 17 April 2022. There was no travel link to an endemic country, and no source of the infection could be identified through extensive backwards contact tracing for 21 days before symptom onset. None of the 98 contacts linked to this household cluster, including healthcare workers, developed monkeypox up to 25 May 2022, although follow-up is still ongoing.

Third, on 16 May 2022, four new confirmed MPXV infections were reported among adult men (aged ≥ 18 years) in England. These new cases had no known links with the earlier cases and had not travelled to a country with risk of MPXV. Two were linked as sexual partners. The incident response was escalated due to suspected community transmission and case definitions more specific to the outbreak developed [7]. An alert was raised among clinical networks leading to some individuals with ulcerative and vesicular rash being recalled to be tested for MPXV, for example in sexual health services (SHSs).

Up to 25 May 2022, 86 cases have been laboratory confirmed in the UK as infected with MPXV. This report describes the data available on UK confirmed cases to up to 25 May 2022. 

## Epidemiology

The outbreak cases are currently grouped into three distinct incidents with one case resident in Scotland and 85 in England. Ninety-one per cent of cases are known to be London residents (69/76 with reported home address), and a small number of cases reside in the East (n = 2), North-East (n = 1), and South-East (n = 4) regions of England. The symptom onset dates of confirmed cases with available information range from 21 April 2022 to 19 May 2022 (Figure). The median reporting delay, calculated as the number of days between onset date and date that the case was recorded in the Public Health case management system, is 11 days for cases up to 25 May (interquartile range (IQR): 6–14 days).

**Figure:**
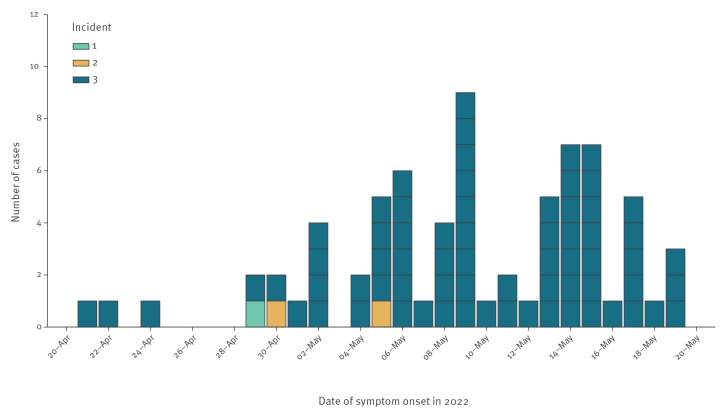
Distribution of laboratory-confirmed monkeypox cases, by symptom onset date and associated incident, United Kingdom, 20 April−25 May (n = 72 with known onset dates)

A total of 82 confirmed cases reported in England were linked to Incident 3. These were neither linked with the first travel-related case (Incident 1) nor the household cluster (Incident 2). Of the cases linked to Incident 3 in England, information on gender was available for 79; all were men. Among these, 66 (83%) cases are known to be gay or bisexual or other men who have sex with men (GBMSM). Sexual orientation and/or behaviour are currently unknown for 16 cases. The median age of Incident 3 cases was 38 years (IQR: 32–43 days). Within the 21 days preceding symptom onset, 18 cases reported foreign travel to multiple countries outside Africa. Investigations are ongoing to determine potential acquisition of infection outside the UK among these cases. Sexual health histories have identified links to sex on premises venues, private sex parties, and the use of geospatial dating apps, both in the UK and abroad. So far, no single factor or exposure that links the cases has been identified.

## Contacts

Contacts of cases linked to Incidents 1 and 2 in this outbreak were mainly passengers on the same flight as the travel-related case or healthcare workers exposed before patients were recognised to be suspected monkeypox cases. In contrast, many contacts in Incident 3 were in the community. There was a median of four and maximum of 25 reported community, close household, or sexual contacts per confirmed case in this incident. This excludes any healthcare worker contacts who were managed by occupational health services in National Health Service (NHS) hospitals. In total in Incident 3, 356 community contacts were identified, in the following settings: 83 household (23%), 78 sexual (22%), 89 friend/shared space (25%), 82 workplace (23%), and 24 community healthcare (7%). Where known, 61% of contacts were male (90/148), 48% were aged 20 to 39 years (46/96), and 33% aged 40 to 59 years (32/96).

In some instances, identifying the true number of sexual contacts was difficult due to contexts like group sex. Of the 78 sexual contacts who were reported in Incident 3, only 28% (n = 22) had names or contact details reported. Several cases declined to share personal details of their sexual contacts, or reported multiple anonymous contacts, such as in dark rooms and cruising grounds. This challenged public health action and secondary attack rate calculations.

## Public health actions

The public health response in the UK has focused on: 

(i) improving early case ascertainment through clear public health messaging and risk communication to populations at higher risk;

(ii) preventing onwards transmission through case isolation and tracing of identifiable contacts;

(iii) offering smallpox immunisation (Imvanex, Bavarian Nordic, Kvistgård, Denmark) to high-risk household and identified close contacts up to 14 days post exposure [8].

(iv) international alerts to the World Health Organization and to the European Union on 07 May for Incident 1, 13 May for Incident 2 and 15 May for incident 3, including posts on the European surveillance portal for infectious diseases (EpiPulse) on 07 and 16 May 2022.

Forward-traced contacts are defined as persons exposed in the period between the case’s symptom onset until the lesions have healed and scabs have fallen off. These contacts are being followed up and are managed according to their level of risk (described in [8]), by vaccination (ideally within 4 days of exposure or up to a maximum of 14 days), either passive or active surveillance and recommending quarantine until 21 days post exposure [8].

Uptake of vaccination among those in the high and medium risk categories is low, with 69% (169/245) of healthcare workers and 14% (15/107) of community contacts, reported to have taken up the offer of vaccination by 24 May (Table). 

**Table:** Description of assessed close contacts of monkeypox cases according to their origin, risk category, and management, United Kingdom, up to 24 May 2022 (n = 588)^a^

Contact type	Low risk Number	Medium risk Number	High risk Number	Total medium and high risk Number	Vaccinated among medium and high risk Number (%)
Community^b^	97	70	37	107	15 (14)
Healthcare^c^	139	197	48	245	169 (69)

NHS: National Health Service.

^a^ Close contacts assigned a risk category up to 24 May 2022.

^b^ Community contacts include household, travel, workplace, shared space and community healthcare contacts.

^c^ Healthcare contacts include healthcare workers in eight NHS hospitals.

UK Health Security Agency (UKHSA) has rapidly worked with professional agencies, such as the British Association of Sexual Health and HIV (BASHH) and the British HIV Association, third sector organisations, including the Terrence Higgins Trust (THT) and Stonewall, apps like Grindr, and organisers of Pride and other relevant events to develop communication and community engagement strategies to support case finding and address stigma.

## Discussion

This is the first reported sustained transmission of MPXV in the UK with evidence of human-to-human transmission through close contact, including in sexual networks. Outbreaks in MPXV endemic countries have been mainly due to zoonotic introductions with subsequent animal-to-human transmission and limited human-to-human transmission [9]. In non-endemic countries, the theoretical possibility that zoonotic outbreaks or imported cases could lead to new areas of endemic disease has been highlighted. The current outbreak in the UK has been followed by cases reported in several countries in Europe, most reporting an overrepresentation of GBMSM among new cases, suggesting transmission in these sexual networks [10]. In Central and West Africa where MPXV circulates endemically, information on person-to-person transmission is limited, however given the expected susceptibility of the global population following the cessation of the smallpox vaccination campaign in the 1970s, spread to non-endemic areas was a recognised risk [11]. The current outbreak signals a change in basic assumptions about the epidemiology of MPXV in Europe with profound implications for surveillance and control.

In the current study, we asked about HIV infection and pre-existing health conditions. We, however, decided not to include this information in this report as the data were still preliminary/incomplete at the time of writing.

While the aim should remain elimination, public health success will require a concerted international effort to: identify cases and their contacts; engage communities on case finding and prevention; and address knowledge gaps including the role of sexual contact in sustaining this outbreak, and whether this is explained by skin-to-skin, respiratory, or sexual transmission. In addition, public health risk assessments and communication will be critical due to the challenges associated with contact tracing in the sexual networks reported by cases. Scaling capacity for routine testing and consideration of alternative approaches to vaccination, including pre-exposure vaccination and wider vaccination for those at higher risk of infection, are essential if rapid outbreak control is not achieved globally.
