# Erratum for Euro Surveill. 2022;27(22)

**DOI:** 10.2807/1560-7917.ES.2022.27.23.220609c

**Published:** 2022-06-09

**Authors:** Eurosurveillance editorial team

**Affiliations:** 1 European Centre for Disease Prevention and Control (ECDC), Stockholm, Sweden

In the article ‘Community transmission of monkeypox in the United Kingdom, April to May 2022’ by Vivancos et al., published on 02 June 2022, the interquartile range (IQR) unit for the age of Incident 3 cases was in days rather than in years, which was incorrect. This was corrected on 08 June 2022. We apologise for any inconvenience this may have caused.

